# Dysregulation of cancer genes by recurrent intergenic fusions

**DOI:** 10.1186/s13059-020-02076-2

**Published:** 2020-07-06

**Authors:** Jae Won Yun, Lixing Yang, Hye-Young Park, Chang-Woo Lee, Hongui Cha, Hyun-Tae Shin, Ka-Won Noh, Yoon-La Choi, Woong-Yang Park, Peter J. Park

**Affiliations:** 1 Department of Health Sciences and Technology, Samsung Advanced Institute for Health Sciences & Technology, Sungkyunkwan University, Seoul, South Korea; 2 Samsung Genome Institute, Samsung Medical Center, Seoul, South Korea; 3 Veterans Medical Research Institute, Veterans Health Service Medical Center, Seoul, South Korea; 4 Department of Biomedical Informatics, Harvard Medical School, Boston, MA USA; 5 Ben May Department for Cancer Research and Department of Human Genetics, The University of Chicago, Chicago, IL USA; 6 Samsung Biomedical Research Institute, Sungkyunkwan University School of Medicine, Suwon, Gyeonggi South Korea; 7 Laboratory of Cancer Genomics and Molecular Pathology, Samsung Biomedical Research Institute, Samsung Medical Center, Seoul, South Korea; 8 Department of Pathology and Translational Genomics, Samsung Medical Center, Sungkyunkwan University College of Medicine, Seoul, South Korea

**Keywords:** Structural variations, Whole-genome sequencing, Chimeric transcripts, Enhancer hijacking

## Abstract

**Background:**

Gene fusions have been studied extensively, as frequent drivers of tumorigenesis as well as potential therapeutic targets. In many well-known cases, breakpoints occur at two intragenic positions, leading to in-frame gene-gene fusions that generate chimeric mRNAs. However, fusions often occur with intergenic breakpoints, and the role of such fusions has not been carefully examined.

**Results:**

We analyze whole-genome sequencing data from 268 patients to catalog gene-intergenic and intergenic-intergenic fusions and characterize their impact. First, we discover that, in contrast to the common assumption, chimeric oncogenic transcripts—such as those involving *ETV4*, *ERG*, *RSPO3*, and *PIK3CA—*can be generated by gene-intergenic fusions through splicing of the intervening region. Second, we find that over-expression of an upstream or downstream gene by a fusion-mediated repositioning of a regulatory sequence is much more common than previously suspected, with enhancers sometimes located megabases away. We detect a number of recurrent fusions, such as those involving *ANO3*, *RGS9*, *FUT5*, *CHI3L1*, *OR1D4*, and *LIPG* in breast; *IGF2* in colon; *ETV1* in prostate; and *IGF2BP3* and *SIX2* in thyroid cancers.

**Conclusion:**

Our findings elucidate the potential oncogenic function of intergenic fusions and highlight the wide-ranging consequences of structural rearrangements in cancer genomes.

## Background

Numerous studies have described somatic structural variations (SVs) in cancer, identifying some rearrangements as tumor drivers that may serve as therapeutic targets in many tumor types [1–5]. Among the examples are *BCR-ABL1* in chronic myelogenous leukemia, for which the tyrosine kinase inhibitor imatinib works efficaciously [1], and *TMPRSS-ERG* in prostate cancer, present in nearly half of the prostate cases and is useful as a diagnostic tool [4]. In these fusions, breakpoints occur at two *intragenic* positions, resulting in in-frame gene-gene fusions that produce chimeric mRNAs [6]. However, among all somatic rearrangements in the genome, the fraction of fusions with two intragenic breakpoints is small. More common are those with one or two *intergenic* breakpoints, which we refer to as “gene-intergenic” and “intergenic-intergenic” fusions, respectively. The impact of such intergenic breakpoints on transcriptome has been unclear.

One possible consequence of an intergenic breakpoint is the upregulation of a gene by a different promoter or enhancer that has been brought to the upstream of the gene. This phenomenon is well known, sometimes referred to as promoter or enhancer “swapping” or “hijacking.” A classic example is the translocation between proto-oncogenes and the *IGH* region in lymphomas, identified in the 1980s by FISH [7]. However, with whole-genome sequencing (WGS) and matched RNA-seq data, there is a potential to systematically discover a broader set of such cases and examine their downstream impact. For example, our recent analysis of chromophobe renal cell carcinomas has uncovered recurrent fusions targeting the *TERT* promoter, with the resulting over-expression of *TERT* [8]. Whereas point mutations in *TERT* promoters have been extensively characterized [9], targeting of the same promoter by SVs was not known prior to these WGS analyses. Another example is in acute myeloid leukemia, where a recurrent intergenic inversion or translocation was found to reposition a *GATA2* enhancer to activate the proto-oncogene *EVI1* [10]. This promoter/enhancer swapping is not uncommon: a recent integrative analysis of whole-genome sequencing and RNA-seq data by the International Cancer Genome Consortium has found that hundreds of genes had altered expressions associated with the presence of a nearby SV breakpoint [11].

In this study, we investigate the role of gene-intergenic fusions in four tumor types (breast, colon, prostate, and thyroid). Most DNA-based fusion analyses to date have focused on those with intragenic breakpoints since those are thought to produce chimeric transcripts. However, our analysis has revealed a mechanism by which a gene-intergenic fusion could result in chimeric oncogenic transcripts. This indicates that a portion of chimeric transcripts will be missed by standard genome-based analysis that searches only for intragenic breakpoints for fusion identification. Although transcriptome analysis will reveal such fusions, most cancer genome studies, especially in clinical applications, do not utilize RNA-seq data. In the second part of the work, we describe a number of novel recurrent gene-intergenic and intergenic-intergenic fusions that do not generate chimeric transcripts but consistently upregulate mRNA of cancer genes by SV-mediated repositioning of distant enhancers. Although many such examples have been found in the past, our analysis reveals that the distant enhancers can be megabases away and that the frequency of such functionally relevant fusion events is greater than previously assumed.

## Results

### Classification of fusion events

We performed SV analysis for 268 WGS samples consisting of 40 breast invasive carcinomas (BRCA), 61 colon and rectum adenocarcinomas (COAD/READ), 47 thyroid carcinomas (THCA), and 120 prostate adenocarcinomas (PRAD), obtained from The Cancer Genome Atlas (TCGA) (Additional file 1: Fig. S1a and Additional file 2: Table S1). We utilized Meerkat, an algorithm we have developed previously for WGS data to identify SVs with nucleotide resolution of breakpoints [12] as well as BreakDancer [13]. Meerkat has performed well in a comparative study of SV algorithms [14], and a combination of these two algorithms has been used in many TCGA studies. A combination of four SV algorithms has been used in recent studies by the International Cancer Genome Consortium, but most algorithms use similar principles and the results should not differ significantly. Across all our samples, we identified 13,698 DNA fusions, formed by a variety of mechanisms, including inversions, deletions, translocations, and tandem duplications.

**Fig. 1:**
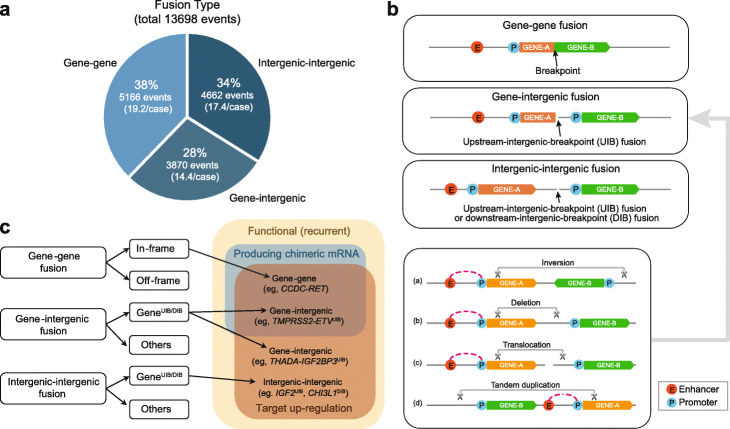
Classification of fusion events. **a** The distribution of different fusion types (gene-gene, gene-intergenic, intergenic-intergenic). **b** Illustrations of the three fusion types. The bottom two cases show a breakpoint upstream of an intact fusion partner. Taking gene-intergenic fusion as an example, we also illustrate how various types of DNA structural variants (inversion, deletion, translocation, and tandem duplication) could result in a gene^UIB^ fusion. **c** Among the recurrent fusions, some produce chimeric mRNAs with increased expression, whereas others upregulate by promoter swapping or other mechanisms without chimeric RNAs. Gene-intergenic fusions with upstream-intergenic-breakpoints (UIB) can produce target upregulation with or without chimeric mRNAs; the mechanism for the generation of chimeric mRNA without a genic breakpoint is described in the text. Intergenic-intergenic fusions can also result in target upregulation. Downstream-intergenic-breakpoint (DIB) cases also occur but are not illustrated here

We classified the fusions into three categories according to the genome annotation of the regions containing the breakpoints and found the following distribution (Fig. 1a): (i) gene-gene, 38%; (ii) gene-intergenic, 28%; and (iii) intergenic-intergenic, 34%. Gene-gene fusions are over-represented (5166 observed, 2740 expected out of 13,698, *p* < 10^−15^ by the proportion test for the null hypothesis that the breakpoints occur at the same frequency across the genome). However, the majority involved fusions with an intergenic breakpoint. Since they were not thought to generate chimeric transcripts, they were set aside in most fusion analyses as being unlikely to be functionally relevant.

Both gene-intergenic and intergenic-intergenic fusions can produce what we refer to as “upstream-intergenic-breakpoint” (UIB) cases, in which the intergenic breakpoint occurs upstream of the second gene (Fig. 1b). They can also produce, to a lesser extent, “downstream-intergenic-breakpoint” (DIB) cases, in which the intergenic breakpoint occurs downstream of a gene. Many types of alteration (e.g., inversion, deletion, translocation, tandem duplication) can give rise to gene-intergenic fusions, as illustrated in Fig. 1b (bottom panel). Gene UIB cases can be further classified into “chimeric-mRNA-producing” (CP) and “non-chimeric mRNA-producing” (N-CP) cases. In Fig. 1c, we summarize the three classes of fusions and their subclassifications, and list representative examples.

Importantly, whereas a “gene-gene” fusion is often assumed to be the cause of a chimeric transcript at the RNA level, we found that it can arise not just from having intragenic breakpoints in two genes (the conventional gene-gene fusion at the DNA level, such as *CCDC-RET* in Fig. 1c) but also from having one intragenic breakpoint and one intergenic one (gene-intergenic fusion at the DNA level, such as *TMPRSS2-ETV* in Fig. 1c). That a gene-intergenic fusion may result in a gene-gene fusion at the RNA level is not obvious at first, but several oncogenic fusions are formed that way, as we show in the following section.

### A mechanism by which gene-intergenic fusions produce chimeric oncogenic mRNAs

We find that a substantial portion of chimeric transcripts is produced by gene-intergenic fusions. In such cases, chimeric transcripts are observed in RNA-seq data, but the presumed intragenic breakpoints are missing in WGS data. In previous studies, the failure to detect an intragenic breakpoint in WGS was often attributed to other factors, such as a low variant allelic fraction, insufficient sequencing depth, or low sensitivity of the detection algorithm. Instead, we find that consistently present in these cases are upstream intergenic breakpoints. To investigate the mechanism behind this phenomenon, we conducted a comprehensive analysis to search for those cases, including a manual review of matched WGS and RNA-seq data (see the “Methods” section).

**Fig. 2:**
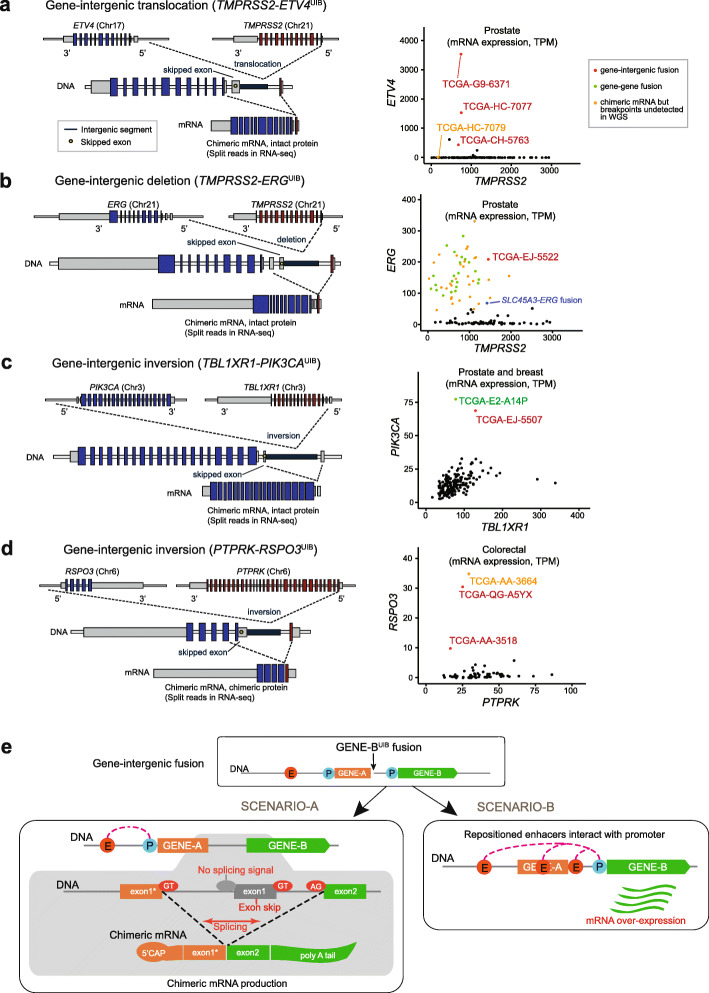
Examples of events that generate gene-intergenic fusions with chimeric mRNAs. **a** In the *TMPRSS2-ETV4*^UIB^ fusion, the breakpoint in *TMPRSS2* occurs in its first intron; the other breakpoint occurs in the upstream intergenic region of *ETV4* by inter-chromosomal translocation. At the mRNA level, the first exon of TMPRSS2 is fused with the second exon of *ETV4* (left), with the skipping of the first *ETV4* exon. The right panel shows the greatly elevated ETV4 expression levels of the three *TMPRSS2*-*ETV4*^UIB^ fusion-positive cases with verified chimeric mRNA (red) compared to other prostate cancer cases (black). In TCGA-HC-7079 (orange dot), the *TMPRSS2-ETV4* chimeric mRNA was identified in RNA-seq, but the exact breakpoints were not identified in WGS due to the low coverage (~ 4–8×) of prostate cases. **b** In the *TMPRSS2-ERG*^UIB^ fusion, the breakpoints are similarly distributed but caused by a deletion to generate fusion transcripts with the first exon of *TMPRSS2* fused with the second exon of ERG. The right panel shows that this case of *TMPRSS2-ERG*^UIB^ showed elevated expression of ERG (red), comparable to the in-frame gene-gene fusion cases of *TMPRSS2-ERG* (green) and *SLC45A3-ERG* (blue). Similar to **a**, orange dots indicate cases with *TMPRSS-ERG* chimeric mRNAs detected in RNA-seq but not in WGS due to the low coverage of this particular tumor type. **c** In one case of *TBL1XR1-PIK3CA* occurring in a prostate sample, the first exon of *TBL1XR1* is fused to the second exon of *PIK3CA* by inversion. In this case, the *TBL1XR1* exon is in the 5′ UTR, and the protein is not chimeric. The other case of *TBL1XR1-PIK3CA* is a gene-gene fusion in BRCA. Both cases showed much greater expression levels of *PIK3CA*. **d** An inversion generates the *PTPRK-RSPO3*^UIB^ fusion, similar to **c**, with the three fusion cases showing markedly increased *RSPO3* expression. **e** Gene-intergenic (gene-gene^UIB^) fusion could produce two different scenarios. In the first scenario (left), the exon before the breakpoint in the upstream gene (GENE-A) and the second exon of the downstream gene (GENE-B) are fused, with the skipping of the first exon of GENE-B due to a lack of splicing signal. The breakpoint typically occurs after the first exon (marked with “*”), but it could occur elsewhere, too. In the second scenario, repositioned regulatory units (e.g., promoters, enhancers, or repressors) result in up- or downregulation of the downstream gene

To illustrate that even well-known chimeric oncogenic transcripts can be produced by gene-intergenic fusions, we show four such instances in Fig. 2: *TMPRSS2-ETV4*^UIB^ (3 cases in PRAD), *TMPRSS2-ERG*^UIB^ (1 case in PRAD), *TBL1XR1-PIK3CA*^UIB^ (1 case in PRAD), and *PTPRK-RSPO3*^UIB^ (2 cases in COAD/READ). The first fusion is formed by translocation, the second by deletion, and the last two by inversion. The chimeric mRNAs from all four fusions were previously reported as having a role in tumorigenesis or tumor progression [4, 15–17], but the possibility that those chimeric mRNAs were formed through gene-intergenic fusions had not been considered. In TMPRSS2*-ETV4*^UIB^, the breakpoint of *TMPRSS2* is located in the first intron and the other breakpoint is located in the upstream intergenic region of *ETV4* due to an inter-chromosomal translocation (Fig. 2a). At the mRNA level, the first exon of *TMPRSS2* is fused with the exon 2 of *ETV4*. In all three prostate genomes with this fusion, the expression level of *ETV4* was elevated greatly compared to the rest of the samples (Fig. 2a, right panel). For *TMPRSS2-ERG*, a well-known fusion in prostate cancer [4], the chimeric mRNA is typically a consequence of two intragenic breakpoints; indeed, we found 25 such cases in our cohort. But we also found an additional case derived from a gene-intergenic fusion (Fig. 2b), which would have been missed by standard DNA analysis alone. The expression of *ERG* in this sample was comparable to that obtained in other cases of gene-gene fusions (Fig. 2b, right panel). Similarly, we found a *TBL1XR-PIK3CA*^UIB^ in a prostate sample (Fig. 2c), in addition to a conventional gene-gene fusion in a breast sample. Finally, we found two cases of *PTPRK-RSPO3*^UIB^, formed by inversions (Fig. 2d). Although the fraction of gene-intergenic fusions generating oncogenic transcripts is small for *TMPRSS2-ERG*, it is likely to be substantially larger for other fusions, given the following counts (Fig. 2): 3/4 (75%) for *TMPRSS2-ETV4*, 1/2 (50%) for *TBL1XR1-PIK3CA*, and 2/3 (67%) for *PTPRK-RSP03*.

Our examination of these cases has revealed how a chimeric oncogenic mRNA can be produced from a gene-intergenic fusion (Fig. 2e): when two genes are juxtaposed in the same strand by an SV event with (i) an intragenic breakpoint for the 5′ gene (“GENE-A” in Fig. 2e) and (ii) the breakpoint of the 3′ gene (“GENE-B”) in the intergenic upstream region, the first part of the 5′ gene before the break and the latter part of the 3′ gene starting at its second exon are fused to form a chimeric mRNA molecule. The first exon of the 3′ gene is skipped because it lacks the canonical AG sequence before the exon that signals the end of intron splicing. Thus, the whole segment between the exon before the breakpoint for the 5′ gene and the second exon of the 3′ gene is regarded as one intron. For the 5′ gene, the fused part is typically the first exon since the breakpoints are concentrated on the first intron. This may be due to the large size of the first introns (Additional file 1: Fig. S1b; note the log scale) as well as a chromatin environment that may be more permissive of a chromosomal break.

In the first three of the four cases (Fig. 2a–c), the first exon of the 3′ gene is in the untranslated region (UTR), without the translational start site. Therefore, for *TMPRSS2-ETV4*^UIB^, for instance, an intact protein-coding region of mRNA for *ETV4* is used with the first exon of *TMPRSS2*. For *PTPRK-RSPO3*^UIB^, the first exon of the 3′ gene does contain the translation start site. In this case, the last exon of *PTPRK* before the breakpoint and the second exon of *RSPO3* are in-frame to produce stable chimeric mRNA transcripts. A previous colorectal cancer study had found recurrent *PTPRK-RSPO3* chimeric mRNAs but without the expected intragenic breakpoints [15]. The non-canonical mechanism we describe provides an explanation. Given that a recent study found that targeting of *RSPO3* in human tumor xenografts with *PTPRK-RSPO3* inhibits tumor growth and promotes differentiation [3], *RSPO3* is a candidate for the treatment of colorectal tumors, and DNA analysis that considers only gene-gene fusions is inadequate for identifying the relevant cases.

Another interesting observation is that most chimeric mRNA-producing gene-intergenic fusions were tumor type- and gene-specific. For example, *TMPRSS-ETV4*^UIB^ fusions were found only in PRAD, while *PTPRK-RSPO3*^*UIB*^ fusions were found only in COAD/READ. Finally, in all these cases, the fusion appears to result in the “borrowing” of the strong promoter and a 5′ portion of the first gene, with subsequent upregulation of the 3′ partner. Consistent with this idea, we find that the expression levels of *ETV4*, *ERG*, *PIK3CA*, and *RSPO3* were elevated ~ 80–800-, ~ 25-, ~ 8-, and ~ 10–30-fold compared to the fusion-negative group of the same tumor type, respectively (Fig. 2a–d).

### Upregulation of intact mRNAs through recurrent gene^UIB^ and gene^DIB^ fusions

We also performed a comprehensive analysis of the cases in which the fusions do not generate chimeric mRNAs but configure the upstream and downstream regulatory sequences—e.g., promoters, enhancers, and insulators—to influence gene expression. A number of such gene-intergenic or intergenic-intergenic fusions have been described in the recent years [14, 18, 19], but they have been limited to analysis of specific genes and with breakpoints occurring close to the gene.

Our initial analysis considered the common scenario in which an intergenic breakpoint is located upstream of a target gene but before the nearest upstream gene. Although this analysis identified a number of recurrent fusions with consistent up- or downregulation of the target genes (*CCND1*, *TOM1L1*, *DDX18*, *UNC5D*, etc.; see the “Methods” section, Additional file 3: Table S2 and Additional file 4: Table S3), our subsequent examination revealed that there are indeed many potentially functional cases in which the breakpoint is further upstream. We therefore increased the distance to potential breakpoints to 4 Mb upstream of the target gene, choosing 4 Mb as a conservative estimate given that a high-resolution Hi-C map has found that 98% of the loops are between the loci that are < 2 Mb apart [20]. In addition, we also included 4-Mb *downstream* region of the target gene, considering that the changes to the downstream enhancers or insulators could also affect the regulation of the target gene. To identify functional UIB and DIB fusions, we imposed strict criteria: (i) recurrence in at least 4 samples and (ii) > 5-fold differential mean expression (and > 4-fold differential expression for each case) of the target gene between the fusion-positive samples and the rest, per tumor type, removing the samples in which the target gene has significant copy number amplification. To ensure that the most likely target gene is selected, our approach considers each gene as a potential target and examines whether it has a set of upstream breakpoints that satisfies the criteria above with strong statistical significance (see the “Methods” section).

**Fig. 3:**
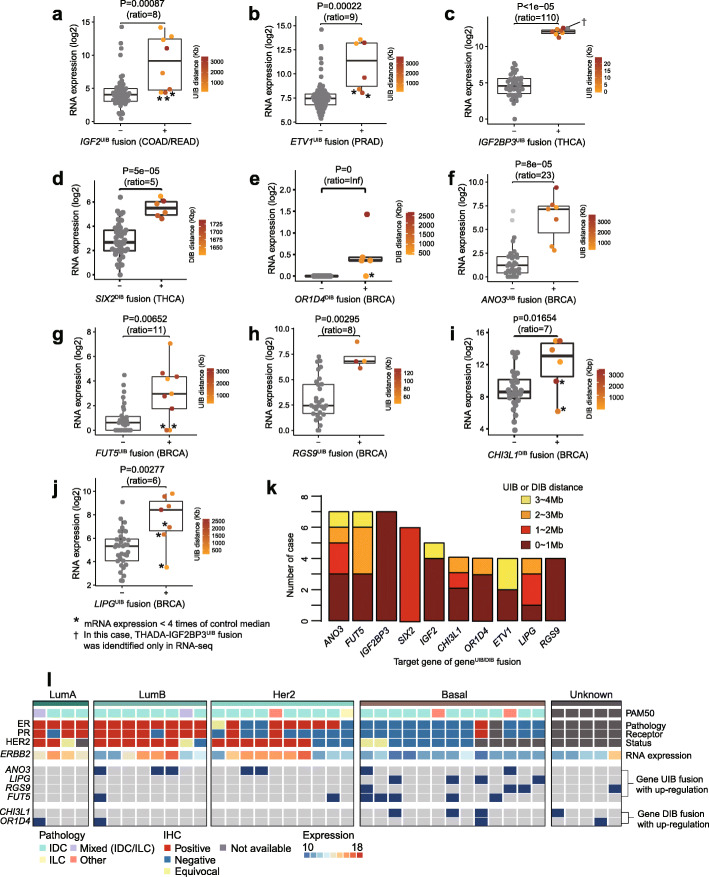
Recurrent gene^UIB^ fusions that massively upregulate target mRNA. **a**–**j** Differential gene expression between the target gene^UIB^ fusion-positive and fusion-negative groups. The ratio between the group means is noted. The BRCA genes are ordered by their expression ratio. Those samples in which the over-expression was less than fourfold compared to the median of the control samples are noted by an asterisk and are excluded from **k** since they are less likely to be functional. Upstream intergenic breakpoint (UIB) distances were described using gradation color in the fusion-positive group. The UIB distance is defined as the base pair distance from the target gene to the upstream intergenic breakpoint. **k** A distribution of UIB distances in 10 genes. Notably, 28% of breakpoints (21/75) were more than 2 Mb away from the putative target gene. **l** The landscape of gene^UIB^ fusion in 40 breast cancer samples. Basal type in PAM50 classification (classifier based on differential expression of 50 genes) is known to assimilate to triple-negative (TN) breast cancer type

This analysis has revealed a number of intact transcripts that are massively upregulated by recurrent gene^UIB^ fusions, including *IGF2* in colorectal, *ETV1* in prostate, *IGF2BP3* in thyroid, and *ANO3*, *LIPG*, *FUT5*, and *RSG9* in breast cancers (Fig. 3, ordered by the expression fold change within a tumor type). Likewise, many were upregulated by recurrent downstream fusions (gene^DIB^), including *SIX2* in thyroid and *CHI3L1* and *OR1D4* in breast cancers (Fig. 3). As shown in Fig. 3k, many are far away from the target gene, with 44% more than 1 Mb away and 23% more than 2 Mb away. *IGF2* and *ETV1* are well-known oncogenes, previously observed with focal amplification in colorectal cancer and as gene-gene fusion in prostate cancer, respectively [21, 22]. There were eight colorectal *IGF2*^UIB^ fusions (8/62, 13%) with an average of 9-fold increase in expression, as well as six *ETV1*^UIB^ fusions in prostate cancer (6/120, 5%) with an average of 9-fold increase. *SIX2*^DIB^ and *IGF2BP3*^UIB^ fusions, with *THADA* as their partner in most cases, are a very interesting case study (Additional file 1: Fig. S2), as described in the next section.

The other six gene^UIB^ and gene^DIB^ fusions were all found in breast cancers (Fig. 3e–j), with recurrence ranging from 4 to 7 per fusion and a resultant change in the expression ranging from 6-fold and up. To determine whether some of them may be driver events in BRCA, we examined the patterns of mutual exclusivity with other tumor characteristics. As shown in Fig. 3l, the *LIPG*^UIB^ and *FUT5*^UIB^ fusions in particular were enriched in the basal type as defined by the 50-gene classifier (PAM50), with *p* values of 0.008 and 0.027, respectively, and were absent in HER2-positive cancers. To examine the consequences of the target gene upregulation, we performed a pathway analysis. For each target gene, we took the 50 samples with the highest expression of that gene from the entire TCGA breast cohort and the 50 with the lowest expression, and then inspected the differentially expressed genes and the enriched pathways (the “Methods” section). For *OR4D1*, *ANO3*, *CHI3L1*, *FUT5*, *LIPG*, *LEP*, *KY*, and *FAM107A*, a large number of genes were coordinately expressed with the target gene (Additional file 1: Fig. S3-S5).

The three particularly interesting genes were *CHI3L1* (chitinase 3 like 1), *FUT5* (fucosyltransferase 5), and *LIPG* (lipase G, endothelial type). *CHI3L1* encodes a 40-kD mammalian glycoprotein, and increased CHI3L1 levels were reported to be correlated with poor prognosis in several types of cancer including breast cancer [23–25]. It was also reported that *CHI3L1* expression is positively correlated with Her-2/new-enriched and basal-like breast cancer [26], but the mechanism behind the expression increase was not established. In our analysis, *CHI3L1*^DIB^ fusion was identified mostly in the basal type, and so we suggest gene^DIB^ fusion as a mechanism for the upregulation of *CHI3L1* in such cases. In our differential expression analysis, we observed dramatic changes in key pathways including cell cycle, regulation of TP53 activity, and EPH-Ephrin signaling (Additional file 1: Fig. S3b). Second, *FUT5* is a member of the fucosyltransferase family and adds fucose to the precursor glycan structures [27]. Liang et al. reported that the *FUT5* expression could be reduced by miR-125a-3p and results in inhibition of proliferation, migration, invasion, and angiogenesis of colorectal cancer [28]. However, no relationship between *FUT5* and oncogenesis was reported in breast cancer, except for one study that suggested an association of *FUT5* expression with poor prognosis in breast cancer multi-cohorts [29]. Interestingly, over-expression of *FUT5* by *FUT5*^UIB^ fusion, present in 17.5% (7/40) of our samples, resulted in changes in various pathways including cell cycle and Notch signaling (Additional file 1: Fig. S5c). Third, upregulation of *LIPG* is intriguing in that it resulted in the alteration of various immune signaling pathways, including TCR signaling, antigen processing, and JAK-STAT signaling (Additional file 1: Fig. S4b). The *LIPG*^UIB^ fusions were enriched in the basal type (described above), and we also found that *LIPG* is highly expressed in the basal type in the larger TCGA cohort (Additional file 1: Fig. S4c, *p* value = 1e−33 by *t* test). In a recent study, the upregulation of *LIPG* was found to be involved in breast cancer cell lipid addiction, thereby contributing to cancer proliferation [30]. These pieces of evidence suggest that *LIPG*^UIB^ fusions may be drivers in basal or basal-like triple-negative breast cancer.

### Upregulation of *IGF2BP3* and *SIX2* by oncogenic fusion of *THADA-IGF2BP3*^UIB^/*SIX2*^DIB^

**Fig. 4:**
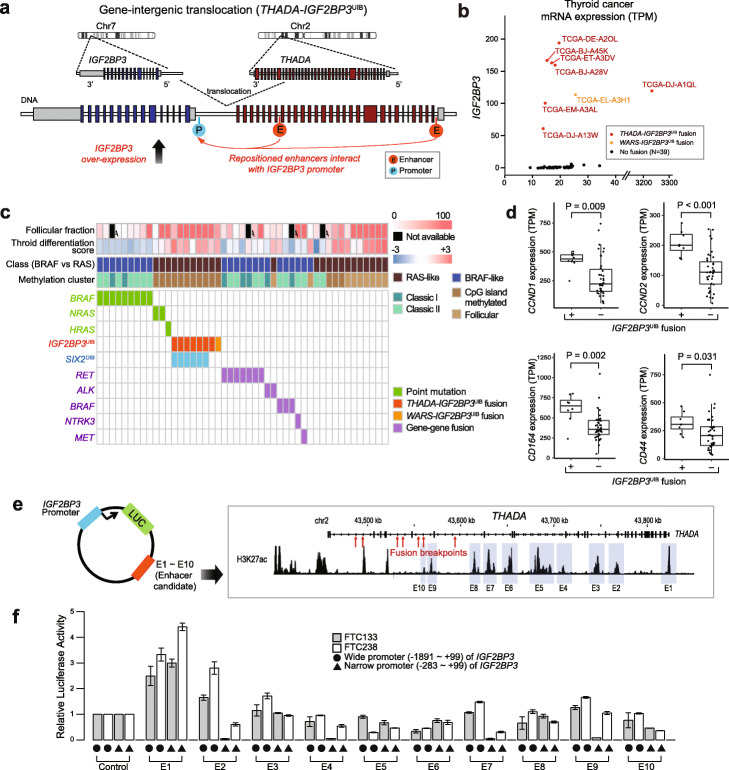
Computational and experimental characterization of *THADA-IGF2BP3*^UIB^. **a** This *THADA-IGF2BP3*^UIB^ fusion was produced as a result of a translocation between chromosomes 2 and 7. RNA-seq data show the upregulation of intact *IGF2BP3* mRNAs, suggesting the repositioning of a regulatory element for *IGF2BP3*. **b** The expression of *IGF2BP3* is dramatically increased for the eight fusion cases (seven *THADA-IGF2BP3*^UIB^ and one *WARS-IGF2BP3*^UIB^). TPM, transcripts per million. **c** The presence of *IGF2BP3*^UIB^ fusion (red) was mutually exclusive with other tumor-driving mutations for all eight cases. The *IGF2BP3*^UIB^ fusion group showed similar molecular and pathologic signatures to *HRAS* or *NRAS* mutation cases, as reflected in high follicular fraction, RAS-like class, CpG island-methylated pattern, and positive thyroid differentiation score. Note that six cases with *IGF2BP3*^UIB^ and *SIX2*^DIB^ fusions were confirmed by whole-genome sequencing. **d***CCND1*, *CCND2*, *CD164*, and *CD44*, which are known to be regulated by *IGF2BP3* in various cancers, are differentially expressed between the 8 *IGF2BP3*^UIB^ fusion-positive and 47 fusion-negative thyroid samples. **e** A vector design (left) to investigate the interaction between the *IGF2BP3* promoter and potential enhancer regions. Ten regions in *THADA* with H3K27ac peaks were tested (right). **f** Dual-luciferase reporter assays using a wide and a narrow *IGF2BP3* promoter in two thyroid cancer cell lines (FTC133 and FTC238) show that two regions (E1 and E2) could interact with the wide promoter and one region (E2) could interact with the narrow promoter. Error bars indicate standard deviations

The gene-intergenic fusion involving *IGF2BP3*^UIB^ (Fig. 4a) was highly recurrent, with a consistent fusion partner. Of the 47 thyroid cases, 7 were *THADA-IGF2BP3*^UIB^ (six cases confirmed by WGS and one by RNA-seq) and the eighth was *WARS-IGF2BP3*^UIB^. The breakpoints in *THADA* (thyroid adenoma associated) occurred between its 29th and 36th introns, whereas those for *IGF2BP3* were 0.4 to 25 kb upstream of the transcription start site (Additional file 1: Fig. S2a). Interestingly, *t* (2;7) involving *THADA* was identified in 2003 by karyotyping in thyroid cancer [31] and has been examined in numerous papers since then—however, all those studies focused exclusively on the downregulation of *THADA*, a gene whose expression is typically high but is reduced by ~ 30–50% upon truncation. The TCGA thyroid paper [32] identified the same fusions but referred to them only as *THADA* fusions; various hypotheses have been put forth in the literature on how the truncated *THADA* from the rearranged allele might be related to the disruption of the apoptosis pathway, but its role has been unclear [33].

Panebianco et al. [34] then discovered that the fusion is an activator of *IGF2BP3*, finding it in 6 out of 21 RNA-seq and 1 out of 4 WGS thyroid cancers. In the TCGA data, we also find that the main target of the fusion is *IGF2BP3*, even though the breakpoints are different: whereas Panebianco et al. [34] reported that *LOC389473* was a hotspot for breakpoints in their thyroid cancer cohort, none of the six *IGF2BP3*^UIB^ in our cohort was located there*.* With more paired DNA/RNA samples and multi-platform profiling in TCGA, we found additional insights. The *IGF3BP3* expression is on average 110-fold greater for the 8 fusion cases compared to the rest (Fig. 4b). To ascertain whether an *IGF2BP3*^UIB^ fusion is a driver event, we cataloged the presence of known driver events in all samples, as annotated in the TCGA thyroid paper [32], such as point mutations in *BRAF*, *NRAS*, or *HRAS* and fusions involving *RET*, *ALK*, *BRAF*, *NTRK3*, or *MET* (Fig. 4c). This analysis shows that the *IGF2BP3*^UIB^ fusion is mutually exclusive with such events. Moreover, those samples with the *IGF2BP3*^UIB^ fusion have distinct molecular and pathologic signatures, with high follicular cell fractions (> 50%), RAS-like features, methylated CpG islands, and positive differentiation scores (Fig. 4c).

To experimentally investigate the mechanism of *IGF2BP3* upregulation, we hypothesized that a *cis*-regulatory enhancer within or around *THADA* would be repositioned to the front of *IGF2BP3* as a result of the *IGF2BP3*^UIB^ fusion. Based on a luciferase assay, we considered both narrow and wide promoters (Fig. 4f, Additional file 1: Fig. S6a and S6b). To determine enhancer locations near *THADA*, we examined the H3K27ac profiles in thyroid and other cell line data from the Canadian Epigenetics, Environment and Health Research Consortium Network [35], and the ENCODE Consortium [36]. We note that the H3K27ac peaks within *THADA* are much stronger than those upstream of *IGF2BP3* in the absence of the translocation (Additional file 1: Fig. S2c), suggesting that the fusion brings a strong set of enhancers upstream of *IGF2BP3*. Selecting ten regions (1 to 1.5 kb) that show strong H3K27ac enrichment upstream of the majority of the breakpoints (Fig. 4e and Additional file 1: Fig. S6c), we performed dual-luciferase reporter assays in two thyroid cancer cell lines FTC238 and FTC133 (Fig. 4e, Fig. 4f and the “Methods” section). From this assay, we identified two regulatory regions that could interact with the *IGF2BP3* promoter: the first one (E1) showed a 2.5–4.5× increase in luciferase activity for interacting with both wide and narrow promoter regions compared to the enhancer-empty control vector; the second one (E2) showed a 1.8–2.8× increase, interacting with only wide promoter region (Fig. 4f). These data show that the recurrent *IGF2BP3*^UIB^ fusion in thyroid cancer is likely to facilitate the interaction between promoter and remote enhancers to upregulate the expression of *IGF2BP3.*

*THADA-IGF2BP3*^UIB^ also affects the expression of other neighboring genes. *SIX2*, which is 1.6 Mb away from the breakpoint in chromosome 2, was consistently upregulated ~ 5-fold in the fusion-positive samples (Fig. 3d, Additional file 1: Fig. S2b). *SIX2* is a transcription factor that contains a homeodomain and plays a key developmental role in the kidney. Recently, *SIX2* was reported to promote cancer metastasis in breast cancer and to attenuate chemotherapeutic sensitivity in non-small cell lung cancer by transcriptional and epigenetics regulation of E-cadherin [37, 38]. *SIX2* also was reported to play a critical role in invasion and drug resistance in colorectal cancer [39]. Although its role in thyroid cancer is unknown, it is noteworthy that *THADA-IGF2BP3*^UIB^ consistently activates not only *IGF2BP3* but also *SIX2*.

In addition, we found that the level of *IGF2BP3* expression is associated with the regulation of various cancer genes including *CCND1*, *CCND2*, *CD44*, *CD164*, *IGF2*, *MMP9*, *PDPN*, and *ABCG2* (Fig. 4d and Additional file 1: Fig. S7), consistent with its role in tumor proliferation, invasiveness, and chemoresistance [40]. Pathway analysis using ConsensusPathDB [41] shows that the 444 genes that have a high correlation (*q* value < 0.05) with *IGF2BP3* mRNA expression were enriched in cancer-related gene sets such as EGFR1, Ras/Raf, sorafenib-related, PI3K-AKT, VEGF/VEGFR, and thyroid hormone synthesis signaling pathways (Additional file 1: Fig. S2d). Based on the molecular signatures, modulating EGFR, Ras, Raf, Akt, and VEGF signaling would be a potential strategy in treating this type of cancer. Panebianco et al. [34] provided experimental evidence that the proliferation of cancer cells with upregulated *IGF2BP3* could be inhibited by the IGF1R inhibitor [34]. With our analysis of TCGA cases showing that the *IGF2BP3* expression in the deceased group (*n* = 16) was significantly higher than that in the alive group (*n* = 484; *p* = 0.035, Wilcoxon rank-sum test), this fusion may serve as a prognostic factor and/or a candidate for therapeutic intervention.

### Prevalence and characteristics of recurrent gene^UIB^ fusions

**Fig. 5:**
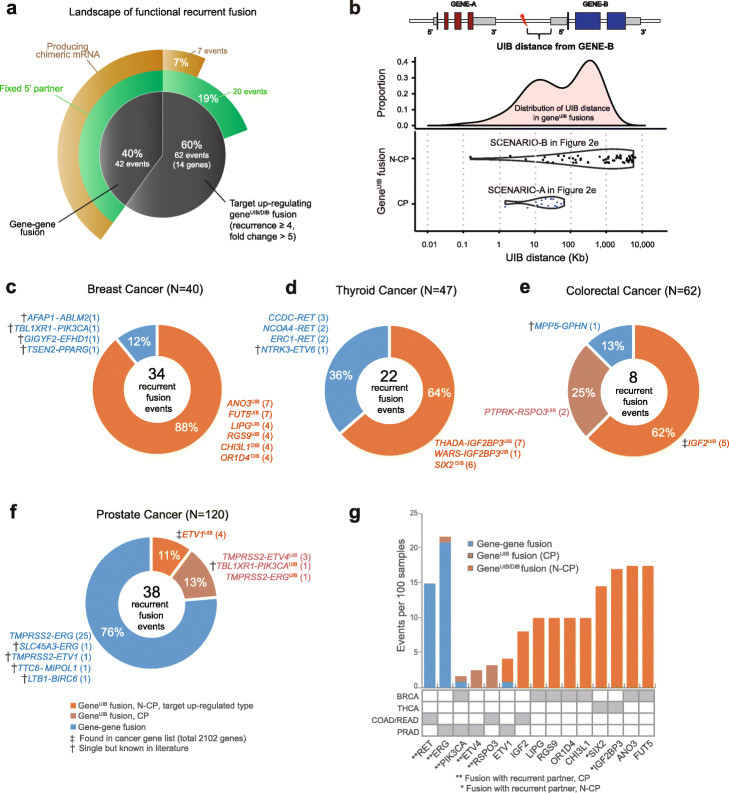
Contribution of recurrent UIB fusions in four tumor types. **a** Proportion of each type of functional recurrent fusions in 268 TCGA samples. Among the target upregulating gene^UIB^ fusions, 32% (20 events, 19% of the total events) had fixed 5′ partners, and 11% (7 events, 7% of the total events) produced chimeric mRNA. **b** The distribution of the distances between the upstream intergenic breakpoints and their target genes. Breakpoints of the fusions that produce chimeric mRNAs (8 recurrent and 9 single cases) are much closer to the target gene (mean, 25 kb; maximum, 64 kb) than in the non-chimeric-producing (N-CP) cases (mean, 1.15 Mb; maximum, 3.7 Mb). Ninety-nine percent of introns are < 100 kb. **c**–**f** Recurrent fusions in BRCA (*N* = 40), THCA (*N* = 47), COAD/READ (*N* = 61), and PRAD (*N* = 120). Genes annotated as cancer-related and singletons in our data but involve a known oncogene are marked with a dagger. **g** Frequency of fusion events per 100 samples and preference for gene-gene vs gene^UIB^ fusions. Fifteen target cancer genes involved in at least two fusion events are shown. Some are chimeric-producing while others are not

Searching for recurrent gene^UIB/DIB^ fusions with massive upregulation of target genes (the “Methods” section), we found 62 such fusion events with downstream impact. Strikingly, this is more than 42, the number of recurrent gene-gene fusion events (Fig. 5a, Additional file 5: Table S4). In 20 recurrent intergenic events, the 5′ partner was also recurrent. In 7 events (Fig. 5a), chimeric mRNA was produced; 9 additional gene-gene^UIB^ fusion events generated chimeric mRNAs but as singletons (Additional file 6: Table S5). Some of these singletons involve cancer-related genes and may turn out to be recurrent if more samples are profiled. For example, in prostate cancer, *TMPRSS2-IRS2*^UIB^ fusion resulted in the upregulation of *IRS2*, an important tumor driver in colon cancer [22, 42]. In the N-CP type, *IGF2BP3*^UIB^ and *SIX2*^DIB^ had inter-chromosomal partners recurrent at the cytoband level, while other UIB events involving *IGF2*, *ANO3*, *FUT5*, *LIPG*, and *CHI3L1* had intra-chromosomal partners recurrent at the cytoband level (Additional file 7: Table S6).

To determine the distinguishing characteristics between the intergenic fusions that produce chimeric mRNAs and others that do not, we examined the distribution of the UIB distances, defined to be the distance between the upstream intergenic breakpoint and the start of the gene. As shown in Fig. 5b, the UIB distances for chimeric-producing cases range from 1.4 to 64 kb, with the mean of 25 kb. We note that these distances are within the range of the intron sizes in the genome, especially for the first introns, which tend to be larger (Additional file 1: Fig. S1b). It therefore seems plausible that the region between the UIB and the gene could be spliced out. In contrast, the UIB/DIB distances for non-chimera-producing fusions range from 0.2 kb to 3.7 Mb, with a mean of 1.15 Mb.

We find that the 14 genes that are significantly upregulated as a result of recurrent gene^UIB/DIB^ fusions are enriched in cancer-associated genes, using a curated cancer gene list (*p* = 0.0017, Fisher’s exact test; see the “Methods” section). This list of ~ 2000 genes we used is the largest of many such lists, although some cancer genes are still missed by the list. In Fig. 5c–f, we list all recurrent fusions in each of the four tumor types. In most cases, except prostate cancer, the number of recurrent, target upregulating intergenic fusions is equal to or larger than that of recurrent gene-gene fusions (Fig. 5c–f). Intriguingly, in BRCA, the number of recurrent target upregulating gene^UIB^ fusions is 30 out of 34 (88%), far higher than the 4 out of 34 (12%) recurrent gene-gene fusions.

Of the genes in Fig. 5a, we summarized the frequency and characteristics of fusions for the 15 recurrently (at least two samples) upregulated target genes (Fig. 5g). Interestingly, chimeric fusions with *ERG*, *ETV1*, or *PIK3CA* could be produced by both gene-gene and gene^UIB^ fusions. On the other hand, several cancer genes, notably *IGF2BP3*, *IGF2*, and *LIPG*, do not produce chimeras but are consistently upregulated by gene^UIB^ fusions. Although most recurrent fusions did not have recurrent partners, the notable exception is *PIK3CA*, *ETV4*, and *RSPO3* among chimera-producing cases, and *SIX2* and *IGF2BP3* among non-chimera-producing cases (Fig.5g, Additional file 1: Fig. S8).

## Discussion

With a joint analysis of WGS and RNA-seq data, we have demonstrated the importance of previously overlooked gene-intergenic and intergenic-intergenic fusions in tumorigenesis. We have described how gene-intergenic fusions could result in the generation of oncogenic chimeric mRNAs with skipping of the first exon of the downstream gene. This mechanism joins the first part of the upstream gene (the last exon before the breakpoint, typically the first exon of the gene) with the second exon of the translocated downstream gene, fundamentally different from the *cis-*splicing described before between adjacent genes by transcriptional read-through [43]. Unfortunately, although we find that chimeric transcripts are more likely to be produced when the breakpoint is sufficiently close (tens of kb) to the downstream gene, this relationship is not exact, and we are not able to make accurate predictions. A further study on other potential factors including chromatin state such as DNA accessibility and DNA methylation will be needed to refine the criteria for chimera production.

We also reported the impact of recurrent (non-chimera-producing) gene-intergenic and intergenic-intergenic fusions in upregulating their target genes, as exemplified by the *THADA-IGF2BP3*^UIB^ fusion and *SIX2*^DIB^ fusion for which integrative analysis identified the correct target. Additionally, *LIPG*^UIB^ and *FUT5*^UIB^ fusions were enriched in basal breast cancer assimilated to triple-negative breast cancers, which is hampered by a lack of a clear understanding of the underlying biology [44]. Importantly, these recurrent gene^UIB^ fusions were at least as frequent as recurrent gene-gene fusions across our four tumor types.

Identifying non-coding mutations that have functional consequences is challenging. For single nucleotide variants/indels, the main approach has been to use epigenetic profiles to identify regulatory elements such as enhancers and then to search for recurrent mutations in those regions. However, distinguishing a tiny fraction of tumor-driving mutations from a sea of background mutations is difficult, especially when recurrence must be defined across a region rather than at a nucleotide position. Identifying driver events in fusion analysis are simpler in some ways, as evidenced by the large number of promising candidate events in our 268 samples. While several recurrent gene^UIB/DIB^ fusions involved known cancer genes, many others—often more recurrent ones—involved genes that were not annotated as cancer-related (Fig. 5g). These should be explored further in future studies. Overall, our results support a growing fraction of tumor-driving events that can be attributed to structural rearrangements.

For clinical applications, panel and exome sequencing are more widespread. Although some rearrangements could be detected from these platforms [45] and panels sometimes contain additional probes across specific introns for common gene-gene fusions, they will miss the majority of functionally important gene-intergenic fusions. Our analysis here thus provides additional motivation for WGS, preferably paired with RNA-seq. Some of the challenges for the application of WGS in the clinical setting include obtaining high-quality DNA/RNA, dealing with tumor-only sequencing data (without the matched normal), accounting for tumor heterogeneity, and filtering a large number of false-positive structural variants when WGS data are derived from formalin-fixed paraffin-embedded (FFPE) tissues. Continued improvements in analytical methods, especially for FFPE data, and a comprehensive database of fusions from a large number of individuals will be indispensable for future efforts.

## Methods

### Sample acquisition, pre-processing, and information

All sequencing data were obtained from The Cancer Genome Atlas (TCGA). Details on sample acquisition, DNA extraction and quality control, sequencing, and other aspects of data generation are described elsewhere [32]. In addition to 268 WGS tumor and normal pairs and matched RNA-seq data, adjacent normal RNA-seq samples from 114 BRCA, 57 COAD/READ, 55 PRAD, and 59 THCA cases were used as controls. For BRCA, COAD/READ, and THCA samples, all WGS data had high (> 30×) coverage. For PRAD, the number of high coverage genomes was only 20; thus, we also included additional one hundred low-coverage (6–8×) samples (some samples sequenced early in the project were done at low coverage). The power to detect SVs from the low coverage data is low; however, we were still able to obtain a large subset of the SVs.

### Characterizing genomic variants

For SVs, we used two algorithms, Meerkat [12] and BreakDancer [13]. For Meerkat, we required at least six discordant read pairs and/or split reads for high-coverage genomes and at least two discordant read pairs and one split read for low-coverage genomes. Variants detected in a tumor sample were filtered by the variants from all normal samples to remove germline events. When both breakpoints of an event fell into simple repeats or satellite repeats, the event was filtered out. A split read had to be aligned uniquely to the predicted breakpoint by BLAT, or the mate of the split read had to be mapped to a position adjacent to the predicted breakpoint. For BreakDancer, the SVs from each tumor sample were filtered by those from its matched normal. The called variants from Meerkat and BreakDancer were combined to increase detection sensitivity. RNA-seq reads were aligned to hg19 using MapSplice [46], and expression values were quantified using RSEM [41]. For SNVs, Mutation Annotation Format (MAF) files were downloaded from the Broad Institute TCGA Genome Data Analysis Center (https://gdac.broadinstitute.org).

### Chimeric mRNA detection from RNA-seq data

To find the chimeric mRNAs, the results from ChimeraScan (v0.4.5) [47] and defuse (v0.6.2) [48] were merged. ChimeraScan uses Bowtie to align paired-end reads to a merged genome-transcriptome reference; deFuse clusters discordant paired-end alignments and predicts fusion boundary with split read analysis. Combining the results from two callers increased detection sensitivity for paired-end data. For single-end RNA-seq data, FusionMap (2015-3-31 version) was used [49]. This algorithm uses a dynamically created pseudo fusion transcript library to accurately map junction-spanning reads. All findings involving known or putative chimera were curated manually after visualizing the reads.

### Computational screening of gene-gene^UIB^ fusion-producing chimeric mRNA

When a gene-gene fusion and a gene-gene^UIB^ (chimera-producing gene-intergenic) fusion produce the same chimeric mRNA, the gene-gene fusion was assumed to be the source of the chimeric mRNA. We screened for the split reads supporting the chimeric mRNAs fused at the predicted exon-exon junctions using an in-house computational method. Briefly, split reads encompassing the exon-exon junctions inferred from WGS-based fusion analysis were extracted from each RNA-seq bam file. Based on the signal-to-noise ratio, we required at least three reads that support the chimera. Each case was visualized and manually verified at the read level [49].

### Computational screening of recurrent gene-gene and recurrent gene^UIB/DIB^ fusion

For each fusion call, intergenic breakpoints were annotated with flanking genes including their location and direction. A gene-gene fusion was considered recurrent when both breakpoints were located within genes and at least two samples shared the gene pair. We investigated recurrent intergenic fusions with two parameter settings. In the initial analysis, a fusion was considered recurrent when at least two samples had an upstream-intergenic-breakpoint between the same target gene and the nearest upstream gene, with consistent mRNA up- or downregulation. Among the 44 genes that are significantly upregulated, there was enrichment for cancer-associated genes as defined by a curated cancer gene list (*p* = 0.00008, Fisher’s exact test; see the last section).

In subsequent analysis, we required at least four samples with the fusion and considered breakpoints within 4 Mb upstream of the gene^UIB^ and downstream of the gene^DIB^. Because breakpoints further away are less likely to be functional and may introduce noise instead, we identified the fusion cases that are most likely to be functional in the following manner. To search the recurrent gene^UIB^ cases, suppose breakpoints are ordered S_1_, S_2_, …, S_*n*_ in the 4-Mb upstream intra-chromosomal region according to the distance from the gene, with S_1_ being the closest. We compared the expression values of all possible groupings S_1_, …,S_*i*_ vs S_*i* + 1_, …,S_*n*_ using the Wilcoxon test and picked the corresponding sample group with the lowest *p* value. If the target gene had > 4-fold upregulation in each of the fusion cases in the group and the mean expression was also > 5-fold greater than the rest of the samples, we considered this fusion to be functional. To minimize the impact of copy number amplification, cases with high-level amplification (the score “2” from putative copy number alterations from GISTIC, in http://www.cbioportal.org) in the target gene were excluded. In Fig. 5, when a fusion occurs once but it produces a previously reported oncogenic chimeric mRNA, we considered it to be part of the “recurrent” set.

### Identifying pathways perturbed in the *IGF2BP3*^UIB^ fusion cases

To determine the impact of the *IGF2BP3*^UIB^ fusion, we identified the genes whose expression was correlated with that of *IGF2BP3* across the 47 thyroid samples. For the 444 genes with a *q* value < 0.05 (|*r*| > 0.627, Pearson correlation), an over-representation analysis (ORA) based on Fisher’s exact test was performed using ConsensusPathDB (http://consensuspathdb.org/), which contained curated interaction networks from genetic, metabolic, gene regulatory, and other interactions [41]. Pathways with *q* values < 0.1 were selected; similar pathways were combined based on the pathway ontology and the gene overlap (overlapping ratio > 0.9). We also performed a Gene Set Enrichment Analysis (GSEA) [50], and the top 20 pathways ranked by normalized enrichment score (NES) were compared with the ORA results. We performed a similar analysis for the target genes of other fusions, using ~ 50 cases with the highest expression of the target gene and ~ 50 cases with the lowest expression. Visualization was performed using an R package, ComplexHeatmap [51].

### Finding the promoter region of *IGF2BP3*

To investigate the promoter region of *IGF2BP3*, six candidate regions were selected around the 5′ UTR. The six regions were (− 1891, + 99), (− 1891, − 1000), (− 1000, + 99), (− 382, + 99), (− 283, + 99), and (− 186, + 99) around the translation start site (ATG). The primers are listed in Additional file 1: Fig. S6a. Among the six, two candidates (− 1891, + 99) and (− 283, + 99) were selected based on their activity in HeLa and FTC238 (Additional file 1: Fig. S6b).

### Luciferase reporter assay for finding enhancers interacting with the *IGF2BP3* promoter

The *IGF2BP3* promoter region was PCR-amplified using Nthy-ori 3-1 (thyroid cell line) genomic DNA as a template and cloned into a pGL3-Basic vector (Asp718/BglII) (Promega). From the H3K27ac profiles obtained from CEEHRC (http://www.epigenomes.ca) and ENCODE (https://www.encodeproject.org), the H3K27ac peaks in all available cell lines, including thyroid cell lines, were examined. Then, PCR-amplified DNA sequences of H3K27ac peak regions in *THADA* (Fig. 4e and Additional file 1: Fig. S6c) were cloned into the designated enhancer cloning position (SalI/BamH1). The primers are listed in Additional file 1: Fig. S6c. FTC238 and FTC133 cells were transfected using Microporator (Digital Bio). Cells were harvested after 48 h, and luciferase activity was measured with the Dual-Luciferase Reporter Assay System (Promega) using MicroplateLuminometers (Berthold). To normalize the transfection efficiency, the pRL-TK plasmid vector (Promega), which carries a Renilla luciferase reporter gene, was co-transfected with reporter construct as described above. All assays were measured in triplicates.

### Cancer gene enrichment analysis and other statistical analyses

To evaluate cancer gene enrichment in target genes dysregulated by gene^UIB^ fusion, we utilized the cancer gene list (2862 genes) curated by the Bushman Laboratory (http://www.bushmanlab.org). After duplicated and non-human genes removed, 2102 genes remained. For up/downregulated target gene sets, Fisher’s exact test was performed. To test mutual exclusivity or co-occurrence between gene^UIB^ fusions and tumor subtypes in breast cancer, the CoMet R package was used [52]. For testing the difference in *IGF2BP3* expression between deceased and live patients, the Wilcoxon test was used. Expression differences in the *IGF2BP3*^UIB^ fusion-positive group and the negative group were tested using the two-sample *t* test. *p* value < 0.05 was considered significant.

## Supplementary information

**Additional file 1: Fig. S1.** Sample information and upstream intergenic breakpoint distances (UIB). **Fig. S2.** Additional analysis of IGF2BP3^UIB^ fusions. **Fig. S3.** Pathway analysis for OR1D4-, CHI3L1- and FUT5-overexpressing breast cancers. **Fig. S4.** Pathway analysis for LIPG-overexpressing breast cancers and LIPG expression in basal and non-basal breast cancer. **Fig. S5.** Pathway analysis for LEP-, KY- and FAM107A-overexpressing breast cancers and FAM107A expression in basal and non-basal breast cancer. **Fig. S6.** Experimental validation. **Fig. S7.** Cancer genes differentially expressed in IGF2BP3^UIB^ fusion-positive vs -negative groups in thyroid cancer. **Fig. S8.** Five gene^UIB^ fusions with fixed 5′ partners.

**Additional file 2: Table S1.** Samples IDs for the 268 TCGA samples.

**Additional file 3: Table S2.** Target up-regulating gene^UIB^ fusions, investigated with the scenario in which an intergenic breakpoint is located upstream of a target gene but before the nearest upstream gene.

**Additional file 4: Table S3.** Target down-regulating gene^UIB^ fusions, investigated with the scenario in which an intergenic breakpoint is located upstream of a target gene but before the nearest upstream gene.

**Additional file 5: Table S4.** Target up-regulating gene^UIB^ fusions, investigated with the scenario in which an intergenic breakpoint is located up to 4 Mb upstream of a target gene.

**Additional file 6: Table S5.** Nine additional gene-gene^UIB^ fusions that generate chimeric mRNAs.

**Additional file 7: Table S6.** Information for gene^UIB/DIB^ breakpoints and partners in cytoband level.

**Additional file 8.** Review history.

## Data Availability

All data used in this paper were generated in The Cancer Genome Atlas and are available from the Database of Genotypes and Phenotypes (dbGaP) with the accession number phs000178.v11.p8 [53]. Access to this dataset requires dbGaP authorization.
