# Supplementary material for: Dysregulation of cancer genes by recurrent intergenic fusions
Source: Genome Biol. 2020 Jul 6;21:166. doi: 10.1186/s13059-020-02076-2 (PMC7339451; doi:10.1186/s13059-020-02076-2)
Supplement: Supplementary file 1 — Additional file 1: Fig. S1. Sample information and upstream intergenic breakpoint distances (UIB). Fig. S2. Additional analysis of IGF2BP3UIB fusions. Fig. S3. Pathway analysis for OR1D4-, CHI3L1- and FUT5-overexpressing breast cancers. Fig. S4. Pathway analysis for LIPG-overexpressing breast cancers and LIPG expression in basal and non-basal breast cancer. Fig. S5. Pathway analysis for LEP-, KY- and FAM107A-overexpressing breast cancers and FAM107A expression in basal and non-basal breast cancer. Fig. S6. Experimental validation. Fig. S7. Cancer genes differentially expressed in IGF2BP3UIB fusion-positive vs -negative groups in thyroid cancer. Fig. S8. Five geneUIB fusions with fixed 5′ partners. [file 13059_2020_2076_MOESM1_ESM.pdf]

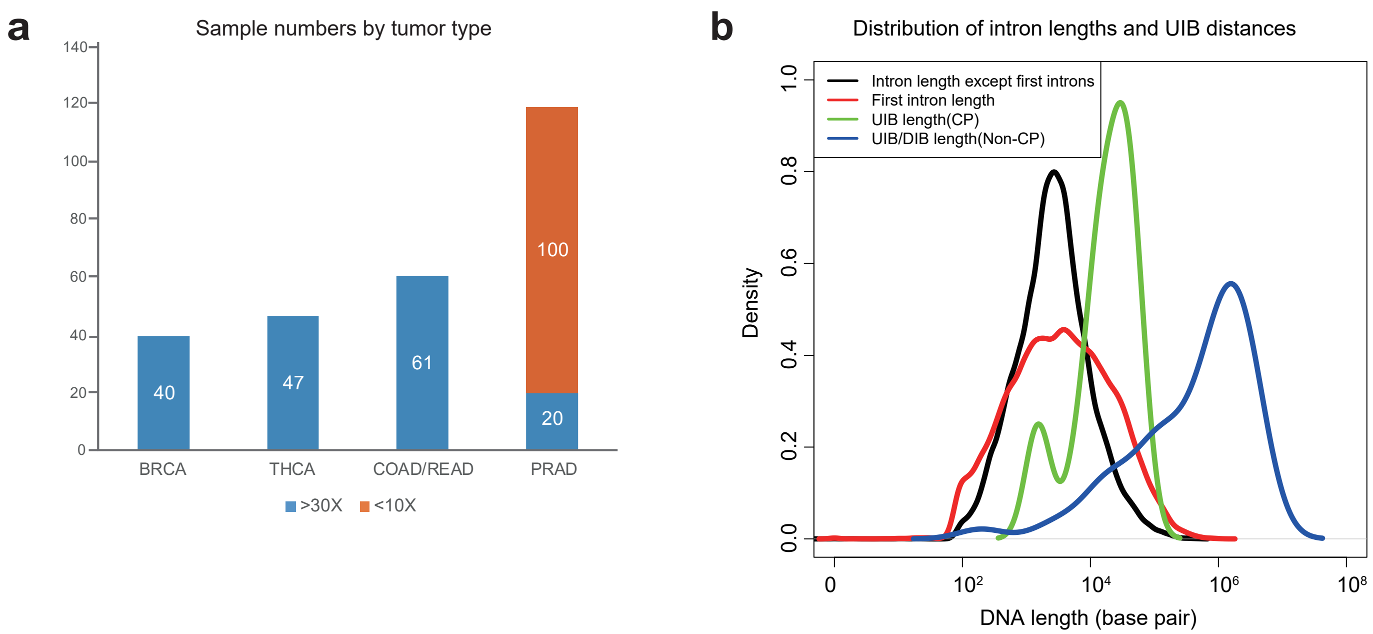

Fig. S1. Sample information and upstream intergenic breakpoint distances (UIB). a. The sample numbers for breast (BRCA), thyroid (THCA), colorectal (COAD/READ), and prostate (PRAD) cancers are shown. The sequencing depth is >30X, except for the 100 PRAD cases (4-8X). Each sample had tumor and normal (blood) WGS pairs as well as matched tumor RNA-seq data. For some samples, WGS from adjacent normal tissues were also available. **b.** The size distribution of all introns except first introns (black), the first introns (red), UIB distances of chimera-producing (CP) gene^{UIB} fusions (green) and Non-CP gene$^{UIB/DIB}$ fusions (blue). A UIB distance is the distance from the upstream intergenic breakpoint to the start of the downstream gene. Note that the distribution of UIB distances of CP type overlaps with that of introns, whereas the distribution of the Non-CP type is far wider (x-axis is log scale).

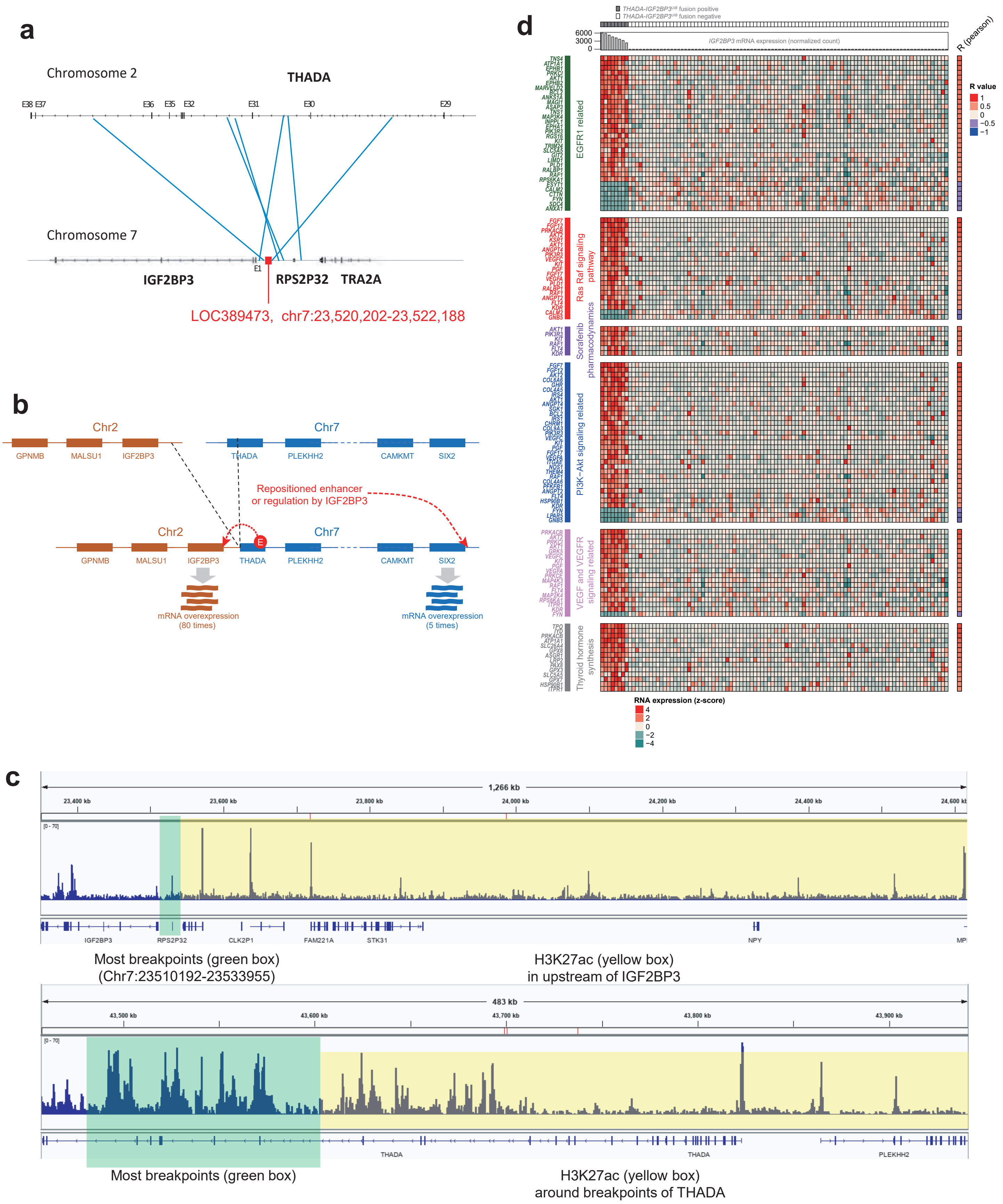

Fig. S2. Additional analysis of *IGF2BP3*^{UIB} fusions. a. Breakpoints in the *THADA* and *IGF2BP3* upstream regions. **b.** A schematic diagram of the *IGF2BP3*^{UIB} fusion neighborhood. **c.** The H3K27ac profile around the breakpoints in the *THADA* and *IGF2BP3* upstream regions in a thyroid cell line. **d.** Pathway analysis of *IGF2BP3*^{UIB} fusion-positive thyroid cancers.

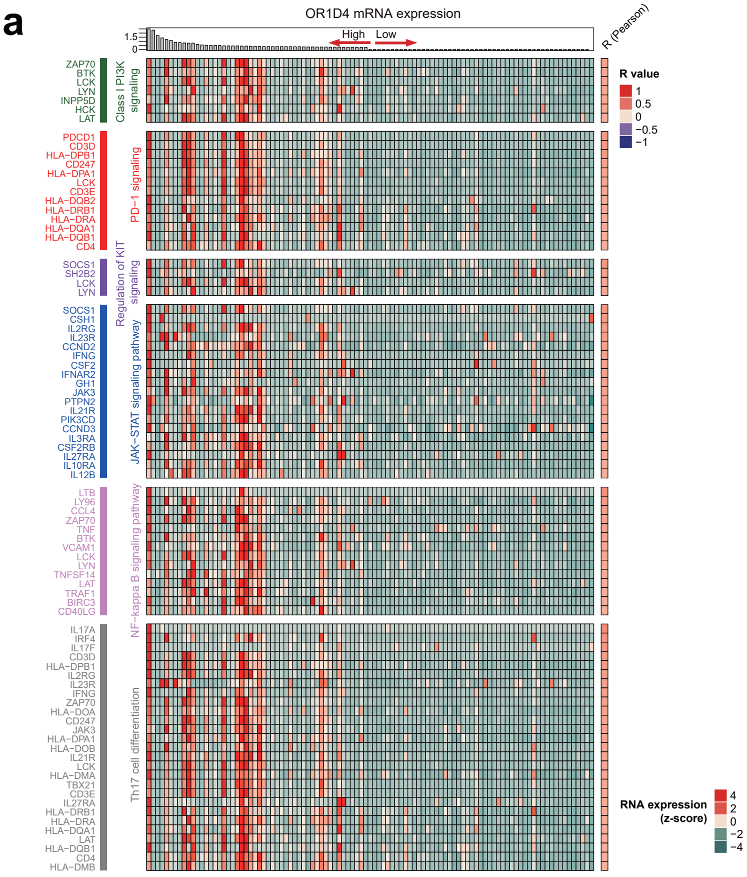

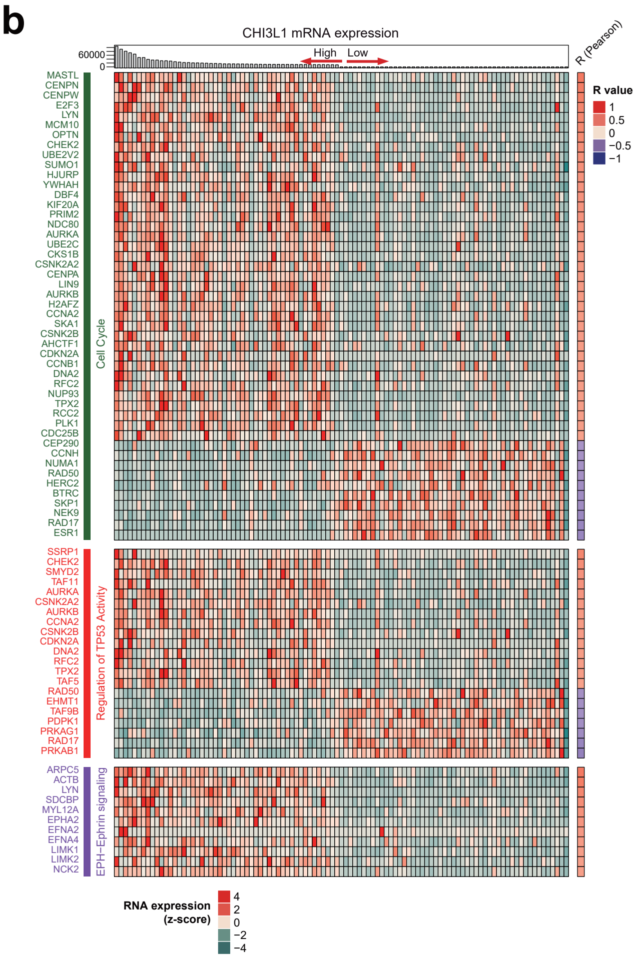

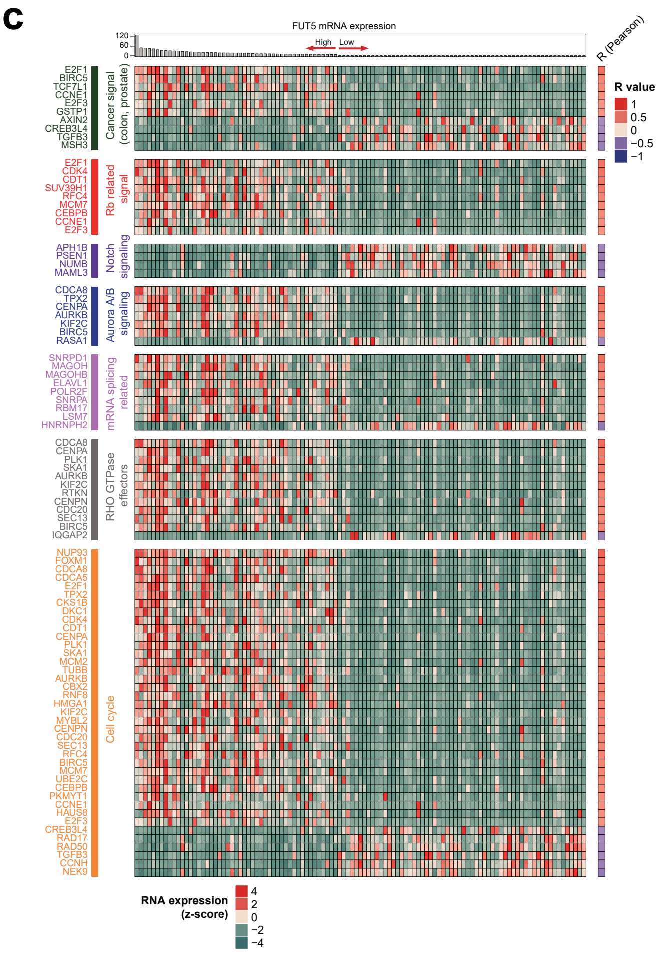

Fig. S3. a. Pathway analysis of *OR1D4*-overexpressing breast cancer. **b.** Pathway analysis of *CHI3L1*-overexpressing breast cancer. **c.** Pathway analysis of *FUT5*-overexpressing breast cancer.

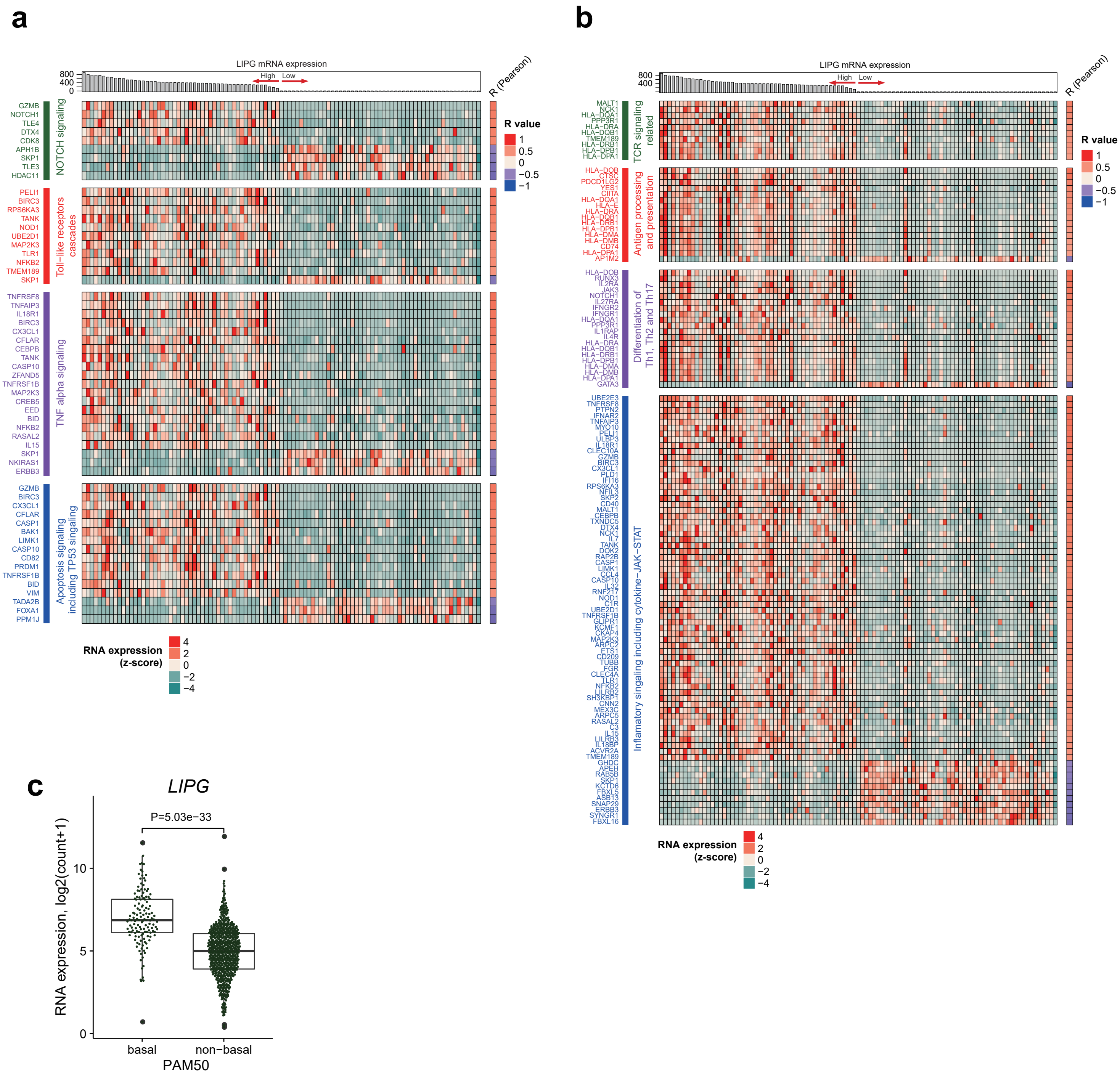

Fig. S4. a. and **b.** Pathway analysis of *LIPG*-overexpressing breast cancer. The left figure (Fig. a.) focused on cancer-related pathways and the right figure (Fig. b.) focused on immune-related pathways. **c.** Differential expression of *LIPG* between basal type and non-basal type breast cancer.

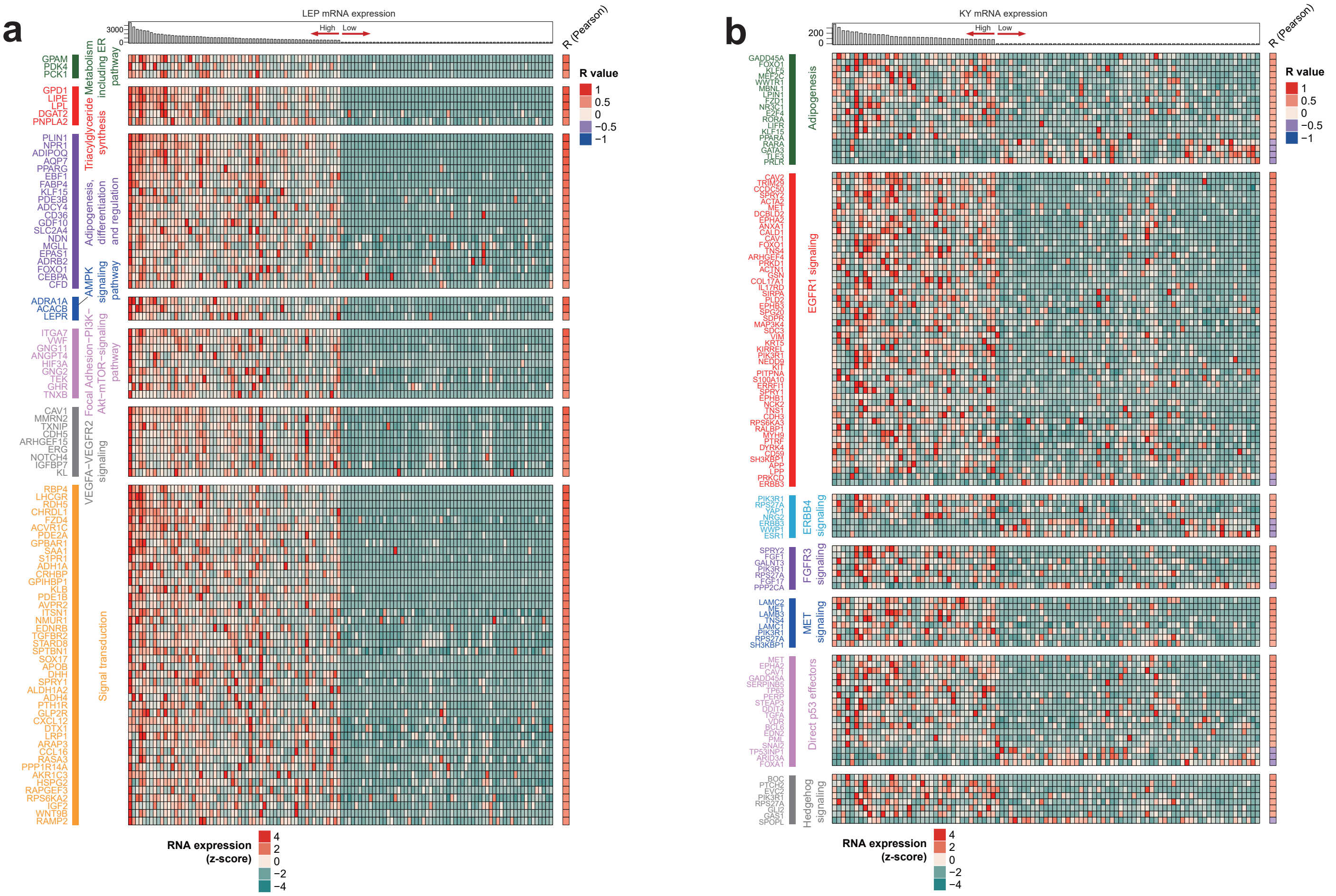

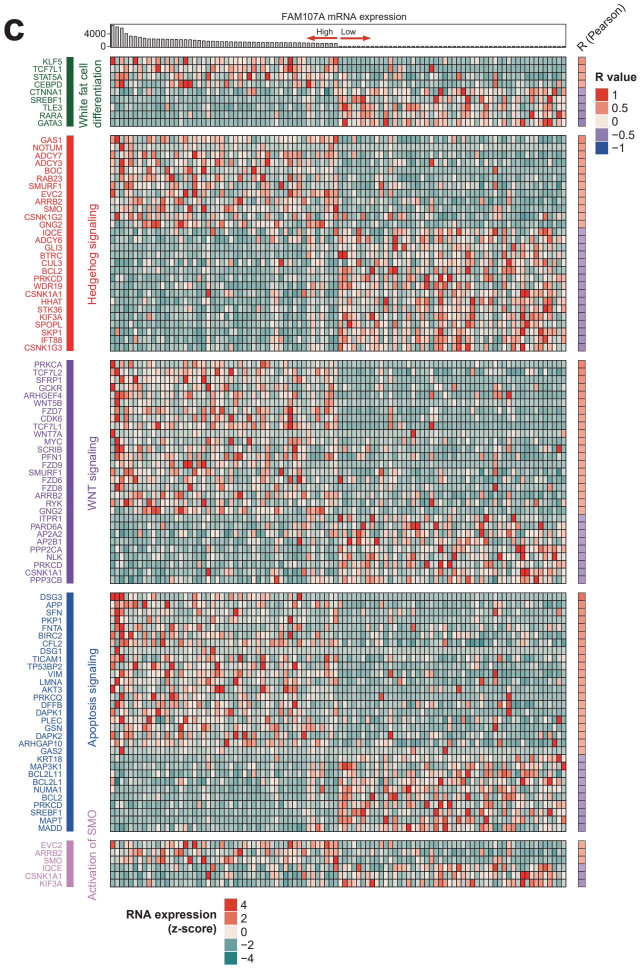

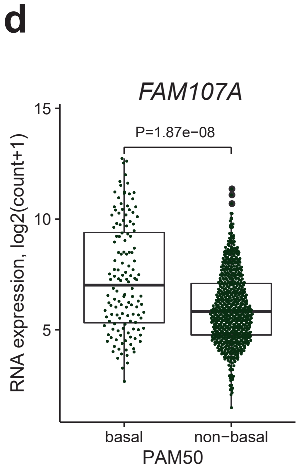

Fig. S5. a. Pathway analysis of *LEP*-overexpressing breast cancer. **b.** Pathway analysis of *KY*-overexpressing breast cancer. **c.** Pathway analysis of *FAM107A*-overexpressing breast cancer. **d.** Differential expression of *FAM107A* between basal type. and

a

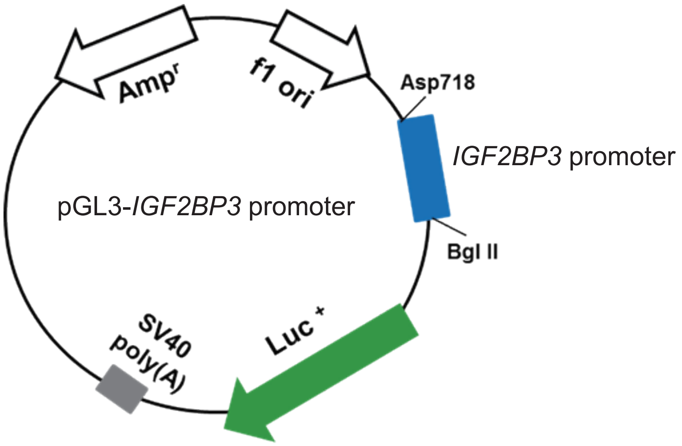

Insert : PCR product (Asp718/Bgl II),
F : -1891 (Asp718) GAC-GGTACC-aaagtaagttccctttaaaagaca
F : -382 (Asp718) GAC-GGTACC-ctctcccaatctcgtttcccctcc
F : -283 (Asp718) GAC-GGTACC-gatttgtagtttgaacacgtggc
F : -186 (Asp718) GAC-GGTACC-ctcccctccctcctgccagtcacc
F : -1000F (Asp718) GAC-GGTACC-gagccttttctgaatcgcgcaacc
R : +99 (Bgl II) GAC-AGATCT-atcagatccgaggcttgttttttc
R : -1000R (Bgl II) GAC-AGATCT-ggttgcgcgattcagaaaaggctc

b

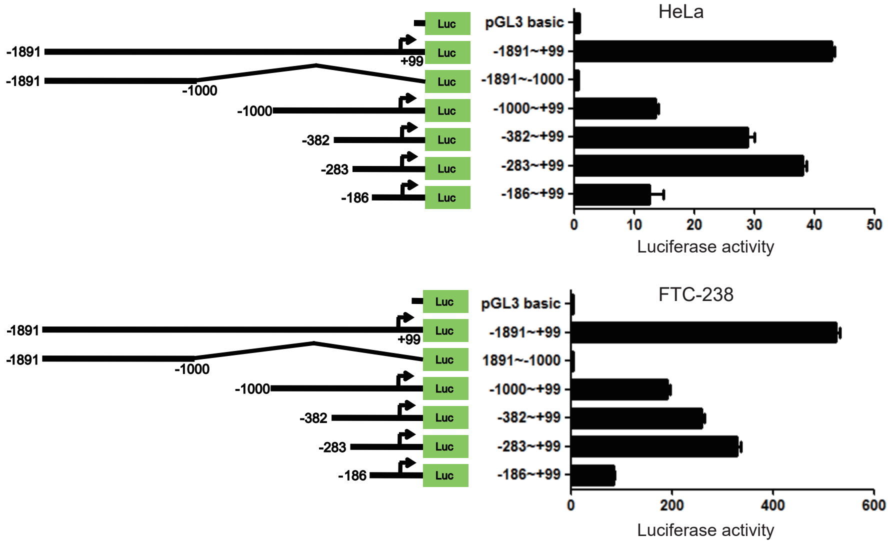

c

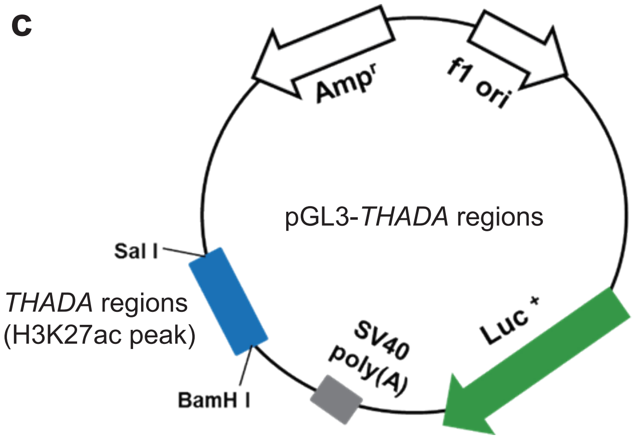

RandomSeq(3bp)-enzymeSeq(6bp)-*THADA*seq(19~20bp)
E1:43,822,500-43,824,000 (1.5Kb)
F(XhoI) -GAC-CTCGAG-GGAATAGAGGCACGCTTCC
R(BamHI)- GAC-GGATCC-TGTGGTAAAGCTATAAAGA

E2:43,746,500-43,748,000 (1.5Kb)
F(SalI) -GAC-GTCGAC-GTGTAGCTATAGATAAATC
R(BamHI)- GAC-GGATCC-GTTCTGATTGGGAAAGTCA

E3:43,683,000-43,684,300 (1.3kb)
F(SalI) - GAC-GTCGAC-AATCCTTCCAGCTAACCAA
R(BamHI)- GAC-GGATCC-GTGTGTGTGTGTGTGTGTG

E4:43,682,000-43,683,300 (1.3kb)
F(SalI) - GAC-GTCGAC-TGAAGGCAATGCTCCATTA
R(BamHI)- GAC-GGATCC-TTTGCACATTTTTAAGACT

E5:43,652,000-43,653,500 (1.5kb)
F(SalI) - GAC-GTCGAC-ACACATGGTTAACCAATCC
R(BamHI)- GAC-GGATCC-GCTCTGTCTTAAGAACATT

E6:43,629,000-43,630,500 (1.5kb)
F(SalI) - GAC-GTCGAC-AAATATCTCCTAGCTAACA
R(BamHI)- GAC-GGATCC-TTAGCTATTATCAGCATCA

E7:43,618,000-43,619,500 (1.5kb)
F(SalI) - GAC-GTCGAC-AATCCCATTTTACCCACAA
R(BamHI)- GAC-GGATCC-GGAAGCAAATGAACATTTA

E8:43,567,500-43,569,000 (1.5kb)
F(SalI) - GAC-GTCGAC-CCTATTAATCTTGCAAAGT
R(BamHI)- GAC-GGATCC-CCATTTGCCTTCTGTGTGG

E9:43,564,683-43,565,659 (976bp)
F(SalI) - GAC-GTCGAC-AGGTCTCTCACGCACTGTT
R(BamHI)- GAC-GGATCC-GGAAATGGGGCATTGTTTA

E10:43,560,000-43,561,500 (1.5kb)
F(SalI) - GAC-GTCGAC–CATCAGCAGTTGGGGGTTG
R(BglII)- GAC-AGATCT–TCCACTTCCCTGTCCAGCT

Fig. S6. Experimental validation. a. The vector design and PCR primer information for the promoter region of *IGF2BP3.* **b.** Regions with the *IGF2BP3* promoter activity in two cell lines (HeLa and FTC238). Note that -1891 to +99 and -283 to +99 showed promoter activity in both cell lines. **c.** The vector design and PCR primer information for investigating the enhancer candidates (H3K27ac peaks) with possible interaction with the *IGF2BP3* promoter.

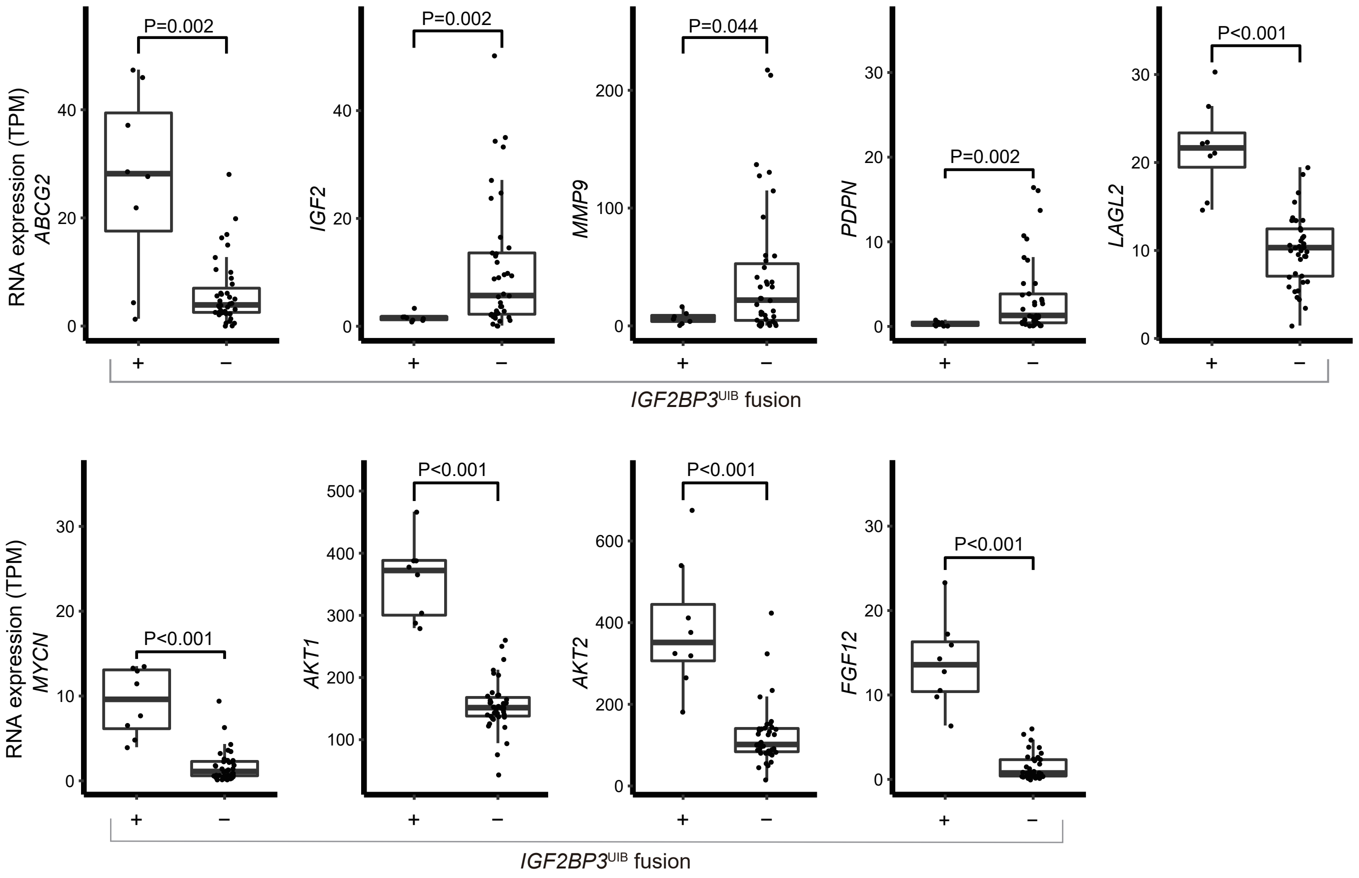

Fig. S7. Cancer genes differentially expressed in *IGF2BP3*UIB fusion-positive vs -negative groups in thyroid cancer. More differential expressed genes in samples with the *THADA-IGF2BP3*UIB fusions; expression levels of *THADA.* We showed in Fig. 4f four genes that were differentially expressed in the 8 *THADA-IGF2BP3*UIB fusion-positive and 47 fusion-negative thyroid cases. Nine additional genes with $p<0.05$ are shown here; all of these have been associated with tumor proliferation, invasion, and drug resistance.

Description	*THADA-IGF2BP3*^{UIB}	*TMPRSS2-ETV4*^{UIB}	*TMPRSS2-ERG*^{UIB}	*PTPRK-RSPO3*^{UIB}	*TBL1XL1-PIK3CA*^{UIB}
3' gene up-regulation	O	O	O	O	O
Producing chimeric RNA	X	O	O	O	O
Type of chimeric RNA breakpoint	-	Coding exon to 5' UTR fusion	Coding exon to 5' UTR fusion	Coding exon to coding exon fusion	5' UTR to 5' UTR fusion
No. of gene-intergenic fusion in WGS	7/47 (THCA)	3/120 (PRAD)	1/120 (PRAD)	2/61 (COAD/READ)	1/120 (PRAD)
No. of gene-gene fusion in WGS	0	0	25/120 (PRAD)	0	1/40 (BRCA)
No. of chimeric mRNA in RNA-seq	0	4/120 (PRAD)	58/120 (PRAD)	3/61 (COAD/READ)	2/160 (PRAD, BRCA)
Type of structural variation	Translocation	Translocation	Deletion	Inversion	Inversion
UIB distance (Kbp)	0.2 ~ 25	6 ~ 28	64	10 ~ 40	15
Reference for chimeric RNA	-	Scott et al<br>Cancer Research, 2006	Scott et al<br>Science, 2005	Somasekar et al<br>Nature, 2012	Nicolas et al<br>Nature Communication, 2014

Fig. S8. Five gene^{UIB} fusions with fixed 5' partners. Details of the five gene-intergenic fusions that have fixed 5' partners. All fusions formed chimeric mRNA, except for *THADA-IGF2BP3*^{UIB}. In the four chimeric-producing fusions, *PTPRK-RSPO3*^{UIB} was an in-frame exon-exon fusion while the other three involved the fusing of a coding exon to the 5' UTR of the target gene (Fig. 2).
