# Supplementary material for: Dysregulation of cancer genes by recurrent intergenic fusions
Source: Genome Biol. 2020 Jul 6;21:166. doi: 10.1186/s13059-020-02076-2 (PMC7339451; doi:10.1186/s13059-020-02076-2)
Supplement: Supplementary file 8 — Additional file 8. Review history. [file 13059_2020_2076_MOESM8_ESM.docx]

Review History

**First round of review**

**This manuscript was previously reviewed at another journal, and the comments were passed along to this reviewer for further assessment.**

**Reviewer 1**

**Are you able to assess all statistics in the manuscript, including the appropriateness of statistical tests used?**

Yes, and I have assessed the statistics in my report.

**Comments to author:**

This manuscript reminds of a recent study by ICGC PCAWG that describes the genomic basis of RNA alterations (PMID: 32025019). Compared to the ICGC study, this study provides more data and insights on intergenic breakpoints, mainly on how they may lead to chimeric RNA transcripts through splicing out of the first exon of the 3'gene, and how they may upregulate gene expression through juxtaposing distant enhancers to the 3' gene. The authors stress such fusion events are typically not well considered in DNA assays, though both can be detected from RNA sequencing data. The finding of THADA-IGF2BP3 rearrangement in thyroid cancer is interesting, and the evidence of its potential driver role is strong. Overall, I find the manuscript is helpful for understanding the contribution of intergenic breakpoints to fusions and gene deregulation. I do have some questions and suggestions as follows.

First, it is unclear to me how they determined the target gene for gene-intergenic and intergenic-intergenic events. The manuscript "considered the common scenario in which an intergenic breakpoint is located upstream of a target gene but before the nearest upstream gene." This scenario certainly makes sense as the nearest downstream gene of the breakpoint is the mostly likely target gene. However, when the breakpoint is further moved up or down up to 4M bps, there might be many other genes residing between the candidate target gene and the breakpoint. How do they pick up the target when so many could be affected by the breakpoint? If by expression, how many genes would show differential expression between fusion-positive and negative samples other than the target gene?

Since the authors suggest gene-intergenic events exert their functional impact through juxtaposing distant enhancers/silencers (such as in the case of THADA-IGF2BP3), it would strengthen the manuscript if they can examine if enhancer epigenetic marks such as H3K27ac have higher/lower signals around the distant breakpoint than the upstream regions of the 3' genes.

**Authors Response**

**Point-by-point responses to the reviewers’ comments:**

*We thank the reviewer for a careful reading of the paper.*

#1. First, it is unclear to me how they determined the target gene for gene-intergenic and intergenic-intergenic events. The manuscript "considered the common scenario in which an intergenic breakpoint is located upstream of a target gene but before the nearest upstream gene." This scenario certainly makes sense as the nearest downstream gene of the breakpoint is the mostly likely target gene. However, when the breakpoint is further moved up or down up to 4M bps, there might be many other genes residing between the candidate target gene and the breakpoint. How do they pick up the target when so many could be affected by the breakpoint? If by expression, how many genes would show differential expression between fusion-positive and negative samples other than the target gene?

*Thank you for your question. You’ve pointed out an important problem that, to us, had no obvious solution. The main issue is that the breakpoints across samples are not usually confined to a narrow interval, as you probably suspected. As a reminder, we pasted Figure 4k on the right. For IGF2BP3, most breakpoints are within 1MB, but for some others, they are spread out further.*

*Therefore, after trying several alternatives, the way we approached this issue was to consider the question starting from each gene. That is, we consider the successive set of upstream breakpoints, starting from the closest one from the gene and including the next closest one at each iteration, and compute the p-value for differential expression for that group of samples with the breakpoints, relative to those whose breakpoints are not included. This approach is repeated for the downstream breakpoints. We found this to be a reasonable heuristic that seemed to work well in practice. In the main text (p.9), we had made a reference to the Methods section:*

*To identify functional UIB and DIB fusions, we imposed strict criteria: (i) recurrence in at least 4 samples; (ii) >5-fold differential mean expression (and >4-fold differential expression for each case) of the target gene between the fusion-positive samples and the rest, per tumor type, removing the samples in which the target gene has significant copy number amplification (see Methods).*

*The relevant subsection in Methods was the following:*

*Computational screening of recurrent gene-gene and recurrent geneUIB/DIB fusion*

*In subsequent analysis, we required at least four samples with the fusion and considered breakpoints within 4Mb upstream of the gene'IB and downstream of the geneDIB. Because breakpoints further away are less likely to be functional and may introduce noise instead, we identified the fusion cases that are most likely to be functional in the following manner. To search the recurrent gene'IB cases, suppose breakpoints are ordered S1, S2, ..., Sn in the 4MB upstream intra-chromosomal region according to the distance from the gene, with S1 being the closest. We compared the expression values of all possible groupings S1,...,Si vs Si+1,...,Sn using the Wilcoxon test and picked the corresponding sample group with the lowest p- value. If the target gene had >4-fold up-regulation in each of the fusion cases in the group and the mean expression was also >5-fold greater than the rest of the samples, we considered this fusion to be functional.*

*We hope the reviewer finds this reasonable. In some cases, there are others genes in the intervening between the breakpoints and a putative target gene, as the reviewer noted, but each of those genes was already tested and found to be less significant. We modified the main text to include the following sentence: “To ensure that the most likely target gene is selected, our approach considers each gene as a potential target and examines whether it has a set of upstream breakpoints that satisfies the criteria above with strong statistical significance (see Methods).”*

#2. Since the authors suggest gene-intergenic events exert their functional impact through juxtaposing distant enhancers/silencers (such as in the case of THADA-IGF2BP3), it would strengthen the manuscript if they can examine if enhancer epigenetic marks such as H3K27ac have higher/lower signals around the distant breakpoint than the upstream regions of the 3' genes.

*We thank the reviewer for the suggestion. Indeed, we examined the H3K27ac profile in the thyroid cell line produced by the Canadian Epigenetics, Environment and Health Research Consortium (CEEHRC; http://www.epigenomes.ca), and found what the reviewer suspected. The H3K27ac peaks within THADA are much stronger than those upstream of IGF2BP3, suggesting that the translocation is likely to have brought strong enhancers to upregulate IGF2BP3. We have added this figure as Supplementary Figure 2c with the following sentence in the main text: “We note that the H3K27ac peaks within THADA are much stronger than those upstream of IGF2BP3 in the absence of the translocation (Supplementary Fig. 2c), suggesting that the fusion brings a strong set of enhancers upstream of IGF2BP3.”*

**Second round of review**

**Reviewer 1**

The authors have addressed my questions. A minor issue is that a recent ICGC paper (PMID: 32024823) should be cited due to its similarity to this study.
